# Tracing the path of disruption: ^13^C isotope applications in traumatic brain injury‐induced metabolic dysfunction

**DOI:** 10.1111/cns.14693

**Published:** 2024-03-27

**Authors:** Charles J. Peper, Mitchell D. Kilgore, Yinghua Jiang, Yuwen Xiu, Winna Xia, Yingjie Wang, Mengxuan Shi, Di Zhou, Aaron S. Dumont, Xiaoying Wang, Ning Liu

**Affiliations:** ^1^ Clinical Neuroscience Research Center, Departments of Neurosurgery and Neurology Tulane University School of Medicine New Orleans Louisiana USA; ^2^ Neuroscience Program, Tulane Brain Institute Tulane University New Orleans Louisiana USA; ^3^ Tulane University Translational Sciences Institute New Orleans Louisiana USA

**Keywords:** ^13^C isotope, bioenergetics, metabolism, traumatic brain injury

## Abstract

Cerebral metabolic dysfunction is a critical pathological hallmark observed in the aftermath of traumatic brain injury (TBI), as extensively documented in clinical investigations and experimental models. An in‐depth understanding of the bioenergetic disturbances that occur following TBI promises to reveal novel therapeutic targets, paving the way for the timely development of interventions to improve patient outcomes. The ^13^C isotope tracing technique represents a robust methodological advance, harnessing biochemical quantification to delineate the metabolic trajectories of isotopically labeled substrates. This nuanced approach enables real‐time mapping of metabolic fluxes, providing a window into the cellular energetic state and elucidating the perturbations in key metabolic circuits. By applying this sophisticated tool, researchers can dissect the complexities of bioenergetic networks within the central nervous system, offering insights into the metabolic derangements specific to TBI pathology. Embraced by both animal studies and clinical research, ^13^C isotope tracing has bolstered our understanding of TBI‐induced metabolic dysregulation. This review synthesizes current applications of isotope tracing and its transformative potential in evaluating and addressing the metabolic sequelae of TBI.

## Graphical abstract

This review provides a comprehensive overview of current research on cerebral metabolic dysfunction and introduces the potential applications of ^13^C isotope tracing in traumatic brain injury (TBI). Future investigations employing the reviewed tracers and others are crucial for advancing our understanding of cerebral metabolic dysregulation following TBI.

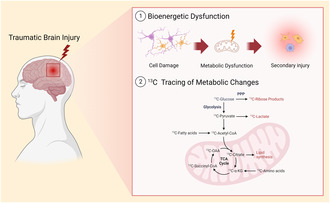

## 1 INTRODUCTION

Traumatic brain injury (TBI) remains a significant public health challenge with limited therapeutic options.^1^ Developing new treatments based on mechanistic rationale is crucial for improving outcomes across diverse TBI patients. Pathophysiological mechanisms post‐TBI can be categorized into primary and secondary injury cascades.^2^ Initiated by primary injury, the secondary injury, which evolves over minutes to months, involves alterations in metabolic and physiologic processes that result in pathologic perturbations including widespread neuroexcitation and ionic flux.^3^

Cerebral metabolic dysfunction, a pathological phenomenon seen in experimental and clinical TBI studies, is a primary contributor to cognitive impairment.^4^ Immediately following primary injury, a transient period of cerebral hyperglycolysis occurs,^5^ followed by a prolonged period of metabolic depression, which largely reflects impaired mitochondrial oxidative metabolism.^5^ Nevertheless, the observed perturbations in glucose utilization implicate alternative energy sources and bioenergetic reprogramming in the recovering brain post‐trauma. Cerebral metabolic dysfunction induces rapid excitotoxicity wherein high extracellular glutamate concentrations disrupt homeostatic ionic gradients and membrane potentials within the parenchyma.^5^, ^6^ This is followed by a cascade of events that includes impaired mitochondrial function, reactive oxygen species (ROS) formation, neuronal apoptosis, and necrotic cell death.^7^ The release of intracellular and intra‐axonal contents from damaged cells can trigger inflammation by activating microglia^8^ and recruiting peripheral immune cells.^9^ In addition, secondary injury caused by metabolic dysfunction also includes blood–brain barrier (BBB) breakdown and edema.^10^

Research efforts to understand TBI‐induced bioenergetic dysfunction may identify targets for novel interventions. A wide variety of experimental approaches have been used to measure metabolic pathways and bioenergetics. ^13^C isotope tracing is a powerful analytic approach that leverages precision quantification techniques like nuclear magnetic resonance (NMR), mass spectrometry (MS), and magnetic resonance spectroscopic imaging (MRSI) in tandem with molecular tracer technology to track the fate of labeled atoms from exogenous isotope‐enriched substrate precursors through to their end metabolic products.^11^ This approach provides precise measurements of substrate flux through key pathways with high temporal resolution in a manner that surpasses alternative research methods, making it highly valuable for investigating metabolic changes in various organ systems and pathologies.^12^, ^13^ Importantly, it has recently shown promise in studying TBI.^14^

This review summarizes existing cerebral metabolic dysfunction research in TBI and introduces the applications of ^13^C isotope tracing in this context. It further underscores this technique's relevance for future TBI research by emphasizing its prior uses in both experimental and human studies to identify areas of focus for future investigations.

## 2 BIOENERGETIC DISTURBANCE IN TBI

The brain is a highly aerobic and energy‐demanding tissue, accounting for only 2% of total body mass while consuming around 20% of the body's daily energy expenditure.^15^ Early bioenergetic crisis is a common pathological occurrence in clinical and experimental TBI studies. The early post‐TBI phase exhibits hyperglycolysis coupled with mitochondrial respiration depression.^16^ Cellular bioenergetics and mitochondrial functions are disrupted after the primary injury, which is particularly evident in the acute phase of moderate to severe TBI.^2^, ^17^ Clinical evidence has established a clear link between cerebral bioenergetics dysfunction and outcomes, especially in moderate and severe TBI cases.^18^ Experimental TBI models reveal a dynamic process of bioenergetic dysfunction, occurring within minutes or hours of injury and persisting for days,^19^ closely tied to injury severity. The energetic crisis contributes to the activation of oxidative and neuroinflammatory stress pathways,^20^ which are critical in driving the secondary brain damage cascades.^21^, ^22^ Importantly, recent findings indicate that bioenergetic disturbances post‐TBI can lead to iron homeostasis dysregulation and increase the susceptibility of cellular membranes to lipid peroxidation due to damaged mitochondria, impaired function of energy‐dependent antioxidant systems, and increased ROS, thereby contributing to the onset and progression of ferroptosis.^23^, ^24^ Thus, the energetic crisis following TBI emerges as a pivotal factor influencing outcomes, with bioenergetic dysfunction serving as a predictor for moderate or severe head injury prognosis.^25^ Understanding the pathological role of bioenergetic crisis in acute secondary brain injury after TBI holds significant importance.

### 2.1 Mitochondrial dysfunction

Traumatic brain injury of all severities affects mitochondrial function.^21^ It has been reported that a substantial decline in mitochondrial respiration is observed as early as 30 min after severe TBI,^26^ reaching peak mitochondrial oxidative damage and dysfunction at 12–24 h.^19^, ^26^ Furthermore, mitochondrial complex 1‐ and complex 2‐driven respiration shows a significant decrease at 1 and 3 h post‐mild, moderate, and severe injuries, with the impact proportional to injury severity.^27^ This underscores that mitochondrial impairment is a central aspect of metabolic dysfunction post‐TBI and precedes various injury cascades, including oxidative stress.^28^

### 2.2 Glucose metabolic dysregulation

Glucose is a primary substrate for generating ATP under physiological conditions which is predominantly achieved through glycolysis and mitochondrial oxidative phosphorylation (OXPHOS).^29^ TBI triggers rapid metabolic dysregulation in the brain through unregulated ion release, mitochondrial damage, and molecular trafficking interruption.^30^ Studies in TBI animal models and head‐injured patients reveal distinct triphasic patterns of cerebral metabolism: hyperglycolysis, metabolic depression, and metabolic recovery.^31^ Hyperglycolysis, marked by increased glucose uptake and lactate concentration, is an immediate response persisting for hours after TBI.^32^, ^33^ Despite its association with enhanced energy production, hyperglycolysis correlates with worse neurological outcomes.^34^ This compensatory mechanism aims to restore the ionic gradient post‐injury^17^ and counterbalance mitochondrial dysfunction.^35^ Following this hyperglycolytic period, cerebral metabolism progresses to a prolonged period of metabolic depression, which features significantly reduced glucose metabolism and robust mitochondrial dysfunction, as documented in TBI experimental models^36^, ^37^ and in humans.^38^, ^39^ The magnitude and duration of glucose metabolic depression is largely dependent on injury severity.^5^ The third and final phase is the metabolic recovery phase. It has been found that the recovery rate of the metabolic function is paralleled with that of neurobehavioral function.^31^ Notably, it has been found that complete recovery is commonly observed in mild TBI patients, but rarely seen after severe TBI.^40^

### 2.3 Alternate fuel sources

#### 2.3.1 Lactate

After severe brain trauma, animal models show a significant rise in brain lactate levels, with immediate and continuous production following severe TBI.^41^, ^42^ This increase is linked to multiple factors, including heightened astrocytic glycolysis due to tissue hypoxia, disrupted neuron–glia metabolic coupling,^43^ activation of astroglia cytosolic malic enzyme,^41^ and upregulation of lactate transporters in endothelial cells.^44^ Excessive lactate accumulation post‐TBI correlates with poor prognosis in animal studies,^41^, ^42^ while low extracellular lactate levels are associated with better outcomes in clinical studies.^45^ Nonetheless, there is growing evidence that lactate metabolism aids cognitive recovery post‐TBI,^46^ acting as a mitochondrial respiration substrate.^47^ This reflects the brain's adaptive response to increased energy demands, highlighting lactate's therapeutic potential for TBI.^48^, ^49^ However, the appropriate timing, dosage, and overall impact of lactate as an alternative energy source post‐TBI are still debated.^50^ The relationship between lactate administration and TBI treatment may depend on the cerebral metabolism's triphasic metabolic pattern, suggesting that lactate administration during the hyperglycolysis phase could worsen outcomes, while its use during metabolic depression and recovery phases could be beneficial. This emphasizes the need for further research to pinpoint the optimal strategies for lactate use in TBI recovery.

#### 2.3.2 Pyruvate

Pyruvate, the product of glycolysis, can be converted into lactate or acetyl CoA. TBI has been found to inhibit both the expression and activity of pyruvate dehydrogenase (PDH)^51^, ^52^, ^53^ in rats, potentially shifting pyruvate metabolism toward lactate. Clinical studies suggest impaired mitochondrial pyruvate metabolism in TBI, leading to decreased aerobic respiration at the location of injury.^54^, ^55^ Elevated lactate‐to‐pyruvate ratio (LPR) in cerebral microdialysis is associated with unfavorable outcomes in TBI patients.^56^ Another human study with 223 TBI patients reported that pyruvate is a significant independent negative predictor of mortality.^45^ Pyruvate supplementation has been shown to improve TBI outcomes.^57^, ^58^

#### 2.3.3 Amino acids

Amino acids, essential building blocks of proteins, can function as alternative substrates for energy production, and their metabolism is reported to be dysregulated in TBI.^59^ Glutamate and glutamine are two important amino acids in the brain with relatively high concentration.^60^ The brain operates under a neuronal and glial metabolism coupling model, where glutamate released by neurons is taken up by astrocytes and converted into glutamine. Conversely, lactate released by astrocytes can shuttle to neurons.^61^, ^62^ TBI leads to neuronal and glial metabolism uncoupling,^63^ which can elicit excitotoxicity.^61^, ^64^ Severe TBI patients also exhibit disturbances in cerebral aspartate metabolism.^65^ Derived from aspartate, N‐acetyl aspartate (NAA) is a brain‐specific metabolite and a sensitive marker of mitochondrial dysfunction and bioenergetics impairment.^66^ A body of evidence has shown that NAA is rapidly and substantially reduced in TBI patients^40^, ^67^ and models.^68^, ^69^ Decreased NAA levels are proportionate to the degree of brain tissue damage post‐TBI, attributed to reduced glucose metabolism and acetyl CoA, coupled with increased acetate metabolism in astrocytes.^67^ Additionally, compromised metabolism of branched‐chain amino acids is linked to cognitive dysfunction after TBI,^70^, ^71^ with evidence suggesting their neuroprotective role.^72^ Dietary supplementation with branched‐chain amino acids has been shown to ameliorate injury‐induced cognitive impairment.^73^

#### 2.3.4 Fatty acids

Fatty acids (FAs) serve as brain energy substrates, and dysregulated lipid metabolism significantly contributes to TBI pathophysiology and worsens neurological outcomes. Clinical studies indicate disturbed lipid metabolism predicts poor outcomes in severely injured trauma patients.^74^ Pre‐clinical investigations suggest dysregulated brain lipid metabolism impairs cognitive performance after TBI.^50^ TBI leads to metabolism dysregulation of lipids and their downstream products, including FAs^75^ and phospholipids.^76^ It has been reported TBI can increase oxidized free FAs,^75^ a prominent product of lipid peroxidation and pro‐inflammatory mediators.^75^ TBI also leads to dysregulation of FA metabolism. For example, TBI reduces l‐carnitine, palmitic acid, and caprylic acid levels, while increasing acylcarnitine levels in the subacute phase.^77^ Medium‐chain FAs, such as octanoic and decanoic acids, increase in severe TBI patients,^78^ potentially influencing the energy crisis associated with mitochondrial failure in TBI.^79^ It is noteworthy that TBI increases FA oxidation to generate ATP in the subacute phase,^77^ which reflects the brain's adaptive response to energy demands by shifting in fuel preference of FAs. In addition, TBI also leads to dysregulation of phospholipid metabolism, which is involved in the aggravation and expansion of neural tissue damage after TBI.^76^, ^80^

#### 2.3.5 Ketone bodies

Ketone bodies, including acetoacetate, β‐hydroxybutyrate, and acetone, serve as natural alternative substrates to glucose for brain energy metabolism.^81^ Shifting toward ketone metabolism can limit the extent of cerebral injury, particularly when glucose utilization is compromised in TBI.^82^, ^83^ Ketone bodies bypass the need for PDH to enter the tricarboxylic acid (TCA) cycle, like when PDH is inhibited in TBI.^52^ Administering exogenous ketones can help spare cytosolic NAD^+^ pools, enhancing metabolic efficiency.^82^ Importantly, ketone metabolism is significantly higher in both the ipsilateral and contralateral sides of the injured brain after TBI.^84^ The energetically favorable mechanisms of ketone body metabolism under TBI conditions help alleviate cerebral energy deficits,^82^ with increased cerebral uptake and oxidation of exogenous β‐hydroxybutyrate improving ATP levels following TBI.^85^

#### 2.3.6 Acetate

Acetate, an oxidizable substance in mitochondria, offers an alternative energy substrate for the brain, potentially aiding in the cellular response to TBI.^86^ Disruptions in conventional energy metabolism in the injured brain may be mitigated by acetate, which is metabolized to acetyl‐CoA by astrocytes. Studies suggest acetate administration may have neuroprotective effects in TBI, improving motor performance and cognitive outcomes and reducing neuronal damage in experimental models.^87^ While acetate metabolism is explored as a therapeutic target for TBI, ongoing research aims to understand its mechanisms, optimal dosages, and potential long‐term effects in clinical settings.

## 3 TRADITIONAL METABOLOMIC TECHNIQUES

Various experimental approaches have enhanced our understanding of the metabolic pathophysiology of TBI in both models and human subjects (Table 1). Techniques such as arteriovenous gradient testing measure cerebral blood flow (CBF) and substrate consumption or release at specific time points following TBI.^25^, ^88^, ^89^ Cerebral microdialysis (CMD) allows in vivo collection of interstitial brain samples and enables focal medication administration.^34^, ^45^, ^90^ Positron emission tomography calculates CBF and cerebral metabolic rates of glucose or oxygen.^39^, ^91^, ^92^, ^93^, ^94^, ^95^, ^96^ MRSI can be used to measure and track the combined extra‐ and intracellular regional concentrations of endogenously labeled substances of interest (e.g. naturally occurring ^1^H, ^13^C, ^15^N, or ^31^P isotope‐containing compounds) and high‐energy phosphate compounds.^54^, ^62^, ^97^, ^98^, ^99^, ^100^, ^101^, ^102^, ^103^ Liquid or gas chromatography (LC/GC)‐MS can measure substances such as proteins and metabolites in various samples including extracellular fluid and brain tissues, among others.^104^, ^105^, ^106^, ^107^, ^108^ A variety of biochemistry assays measure substances (e.g. proteins and metabolites) or enzymatic activities.^52^, ^109^, ^110^, ^111^ Seahorse XF Analyzers can probe mitochondrial oxidative phosphorylation, glycolysis, and overall ATP production in live cells or tissues.^112^ Lastly, fluorescence microscopy with biosensors can measure specific compounds such as NAD^+^/NADH and cAMP.^113^, ^114^ These technologies have been instrumental in establishing our understanding of cerebral metabolic disturbances in TBI. However, a major limitation of most traditional methods lay in that these technologies aren't capable of probing the granular details of complex pathways as they lack the resolution to study substrate turnover across time points.^115^

**TABLE 1:** Summary of traditional research methods for studying energy metabolism in TBI with example studies.

Methods	Principle	Utility in TBI	Animal studies	Clinical studies
Arteriovenous gradient	Invasive test that compares arterial and venous concentrations of metabolic substrates to calculate net consumption/production across a given period	Calculation of CBF; Calculation of cerebral substrate consumption (i.e. oxygen, glucose, lactate, etc.)		Glenn et al. 2003^25^; Holbein et al. 2009^155^; Jalloh et al. 2013^88^; Cardim et al. 2022^89^
Cerebral microdialysis	Invasive technique where extracellular microdialysate containing analytes of interest is collected from the parenchyma via a catheter	Sampling extracellular fluid for measurement of substances (i.e. lactate, pyruvate, glucose, etc.); Intra‐parenchymal infusion of substrates	Fukushima et al. 2009^90^; Krishnappa et al. 1999^156^; Zweckberger et al. 2011^157^	Vespa et al. 2003^34^; Timofeev et al. 2011^45^; Yokobori et al. 2011^158^; Hutchinson et al. 2009^95^; Jalloh et al. 2017^145^; Quintard et al. 2016^49^
Autoradiography and positron emission tomography	Noninvasive test where radiolabeled glucose is administered and imaging measures glucose consumption/sequestration in vivo	Calculating and assessing CBF and cerebral metabolic rate of glucose or oxygen	Sokoloff et al. 1977^91^; Sunami et al. 1989^92^; Moore et al. 2000^93^	Wu et al. 2013^94^; Hutchinson et al. 2009^95^; Kato et al. 2007^96^; Bergsneider et al. 2000^39^
Magnetic resonance spectroscopic imaging	Noninvasive test where endogenous ^1^H, ^13^C, or ^31^P metabolic isotopes can be detected in vivo and quantified using nuclear magnetic resonance technology	Measurement and tracking of the combined extra‐ and intracellular regional concentrations of substance and high‐energy phosphate compounds	Vink et al. 1987^97^; Germano et al. 1988^98^; Millet et al. 2018^99^	Kaibara et al. 1999^100^; Ashwal et al. 2000^101^; Marino et al. 2007^102^; Stovell et al. 2018^103^
Liquid/gas chromatography–mass spectrometry	Noninvasive technique where sample molecules are stratified by size before being identified by fragmentation pattern and quantified	Measurement of substances (i.e. proteins, metabolites) in samples including extracellular fluid, brain tissues, blood, etc	Haskins et al. 2005^104^; Chen et al. 2018^105^	Haqqani et al. 2007^108^; Carmeron et al. 2012^107^
Biochemical assays	Use biochemistry assays to identify and quantify different substrates in a test sample including proteins, enzymes, metabolites, etc	Measurement of substances (i.e. proteins, metabolites) or enzymatic activity in samples including extracellular fluid, brain tissues, blood, etc	Kilbaugh et al. 2015^109^; Lazzarino et al. 2019^52^; Opi et al. 2007^110^; Garik et al. 2018^111^	
Seahorse analyzers	Invasive technique where OCR and ECAR of live cells or tissues are used to interrogate key cellular functions such as mitochondrial respiration and glycolysis	Measurement of mitochondrial respiration, glycolysis, ATP production, or other energy substrates of brain mitochondria, cells, or tissue	Fried et al. 2014^112^	
Fluorescence imaging microscopy	Invasive technique that combines fluorescence microscopy with biosensors to measure substrates in tissue ex vivo	Measurement of cerebral concentrations of substrates including NAD^+^/NADH, ATP, D‐glucose, etc	Díaz‐García et al. 2017^113^; Trevisiol et al. 2017^114^	

## 4 ISOTOPE TRACING

**FIGURE 1:**
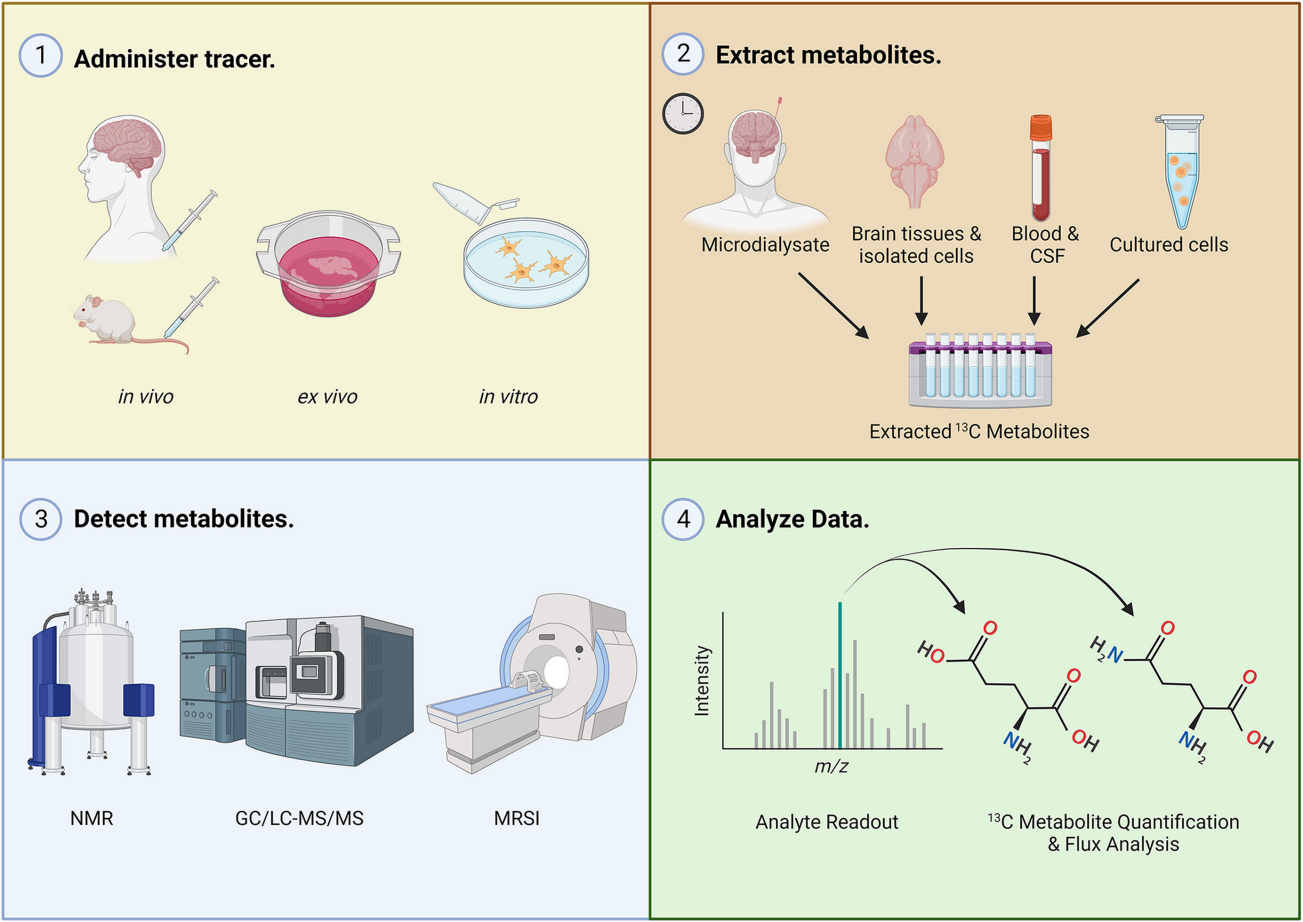
Overview of ^13^C isotope tracing of metabolism in TBI. ^13^C tracer can be administered in vivo to animals or human subjects, ex vivo to fresh tissue, or in vitro to culture model systems (1). Metabolites are extracted from the target tissues or fluids or cells at specified intervals post‐injury (2) and detected using analytic techniques including NMR, mass spectrometry, and MRSI (3). Finally, analyte readouts are analyzed and referenced to metabolite libraries to quantify pathway flux (4). MRSI, magnetic resonance spectroscopic imaging; NMR, nuclear magnetic resonance.

While traditional metabolic measurement techniques focus on net substrate concentrations at specific time points, isotope tracing can infer metabolic flux over a defined period, measuring substrate flow through a pathway per unit of time.^116^ This technology, proven effective in various models and organ systems,^11^, ^12^, ^117^, ^118^ is crucial for characterizing the intricate chemical changes contributing to secondary brain injury (Figure 1, Tables 2 and 3).

**TABLE 2:** Summary of studies using ^13^C isotope tracing to assess metabolic response to TBI in model systems.

Substrate	Tracer(s)	Study	Model	TBI model	Tracer route	Measurement method	Pathway(s) studied	Primary metabolite isotopomer(s) analyzed	Main conclusions
Glucose	1‐^13^C‐glucose	Clausen et al. 2011^123^	Rat	CCI	CMD	GC–MS	Glycolysis, LDH, & MPD	^13^C‐glucose, ^13^C‐lactate, & ^13^C‐glycerol	Glucose is anaerobically metabolized via glycolysis following TBI and increased glycerol levels are primarily from cell membrane degradation
	1‐^13^C‐glucose (w/1,2‐^13^C_2−_acetate)	Bartnik et al. 2010^86^	Rat	FPI	IV	^13^C NMR & ^1^H NMR	Glycolysis, TCA, LDH, & GGS	^13^C‐lactate, ^13^C‐glutamate/glutamine, & ^13^C‐pyruvate	Astrocytes exhibit less impairment of oxidative glucose metabolism than neurons following trauma and are integral for maintaining glutamate levels following injury
	1‐^13^C‐glucose (w/3‐^13^C‐lactate & 2‐^13^C‐acetate)	Lama et. 2014^41^	Rat	Weight drop	IV	^13^C NMR & ^1^H NMR	TCA	^13^C‐lactate, ^13^C‐alanine, ^13^C‐acetate, ^13^C‐NAA, ^13^C‐GABA, ^13^C‐glutamate/glutamine, ^13^C‐succinate, ^13^C‐creatine, ^13^C‐taurine, & ^13^C‐inositol	Several key bioenergetic pathways that link neuronal and glial metabolism under homeostatic conditions become uncoupled within minutes of TBI
	1,2‐^13^C_2_‐glucose	Bartnik et al. 2005^131^	Rat	CCI	IV	^13^C NMR	Glycolysis, TCA, LDH, & PPP	^13^C‐lactate, ^13^C‐glutamate/glutamine, & ^13^C‐pyruvate	Though oxidative metabolism of glucose is maintained, increased PPP activity indicates an increased need for reducing equivalents early after TBI
		Bartnik et al. 2007A^124^	Rat	CCI	IV	GC–MS	Glycolysis & TCA	^13^C‐glutamate	Glucose utilization is primarily anaplerotic in the first 24 h after TBI and then becomes anabolic/regenerative at later time points in the acute phase
		Bartnik et al. 2007B^125^	Rat	FPI	IV	^13^C NMR	Glycolysis, TCA, LDH, GGS & PPP	^13^C‐lactate, ^13^C‐glutamate/glutamine, & ^13^C‐pyruvate	Increased PPP activity indicated the need for reducing equivalents to respond to free radical stress and replenish intracellular NADPH stores in the first 24 h post‐TBI
		Shijo et al. 2017^127^	Rat	CCI	IV	^13^C NMR	Glycolysis, TCA, LDH, & PPP	^13^C‐lactate, ^13^C‐glutamate/glutamine, & ^13^C‐GABA	Exogenous pyruvate and glucose administration enhanced lactate labeling and restored astrocyte PC activity, supporting the role of astrocytes in modulating cerebral glucose metabolism post‐TBI

	1,6‐^13^C_2_‐glucose	Robertson et al. 2013^128^	Rat	CCI	IP	^13^C NMR & ^1^H NMR	TCA & GGS	^13^C‐glutamate/glutamine & ^13^C‐GABA	TBI leads to decreased glutamate production in the injured immature brain at 24 h after TBI, though overall glucose metabolism recovers at 24 h after TBI
		Scafidi et al. 2009^126^	Rat	CCI	IP	^13^C‐NMR	Glycolysis, TCA, & MAS	^13^C‐glutamate/glutamine & ^13^C‐GABA	Oxidative metabolism of glucose is reduced in neurons and astrocytes in the first 6 h after TBI, but the accumulation of related metabolites at later timepoints suggests impaired MAS activity
U‐^13^C_6_‐glucose (w/U‐^13^C_5_‐glutamine)	Liu et al. 2023^33^	PMG culture	Needle scratch	Media	LC–MS/MS	Glycolysis, glutaminolysis, & TCA	^13^C‐fructose‐1,6‐bisphosphate, ^13^C‐phosphoglycerate, ^13^C‐lactate, ^13^C‐citrate, ^13^C‐αKG, & ^13^C‐malate	Cultured primary microglia exposed to damaged neurons exhibit hyperglycolysis and elevated reductive glutaminolysis that lead to pro‐inflammatory activation
β‐HB	2,4‐^13^C_2_‐β‐HB	Scafidi et al. 2022^84^	Rat	CCI	IV	^13^C NMR & ^1^H NMR	TCA	^13^C‐glutamate/glutamine, ^13^C_2_‐GABA, & ^13^C‐aspartate	Neurons and astrocytes can utilize ketone bodies to fuel the TCA following TBI by bypassing PDH in the immature brain
Pyruvate	1‐^13^C‐pyruvate	DeVience et al. 2017^54^	Rat	CCI	IV	MRSI	TCA & LDH	^13^C‐lactate & ^13^C‐bicarbonate	Demonstrated the ability of MRSI and labeled pyruvate to detect cerebral metabolic disturbance in a model of TBI
		Guglielmetti et al. 2017^122^	Rat	CCI	IV	MRSI	TCA & LDH	^13^C‐lactate & ^13^C‐pyruvate	Microglial depletion resulted in greater PDH activity and lower LPR at 7 days after TBI
		DeVience et al. 2021^53^	Rat	CCI	IV	MRSI	Glycolysis & LDH	^13^C‐lactate & ^13^C‐bicarbonate	The level of bicarbonate and the bicarbonate‐to‐lactate ratio decreased on the injured side of the brain 4 h after injury and continued to decrease to 7 days
		Hackett et al. 2023^136^	Rat	CCI	IV	MRSI	Glycolysis TCA, PDH, & LDH	^13^C‐lactate & ^13^C‐bicarbonate	Cerebral bicarbonate production peaks 24 h after TBI and returns to baseline by 10 days post‐injury
	1‐^13^C‐pyruvate (w/^13^C‐urea)	Chaumeil et al. 2023^137^	Mouse	rCHI	IV	MRSI	PDH & LDH	^13^C‐lactate, ^13^C‐pyruvate, & ^13^C‐urea	Changes in cerebral metabolism of pyruvate can be detected more than 3 months after repetitive TBI

Abbreviations: CCI, controlled cortical impact; CMD, cerebral microdialysis; FPI, fluid percussion injury; GABA, γ‐aminobutyric acid; GC–MS, gas chromatography with mass spectrometry; GGS, glutamate‐glutamine shuttle; IP, intraperitoneal; IV, intravenous; LC–MS/MS, liquid chromatography with tandem mass spectrometry; LDH, lactate dehydrogenase; LPR, lactate/pyruvate ratio; MAS, malate–aspartate shuttle; MPD, membrane phospholipid degeneration; MRSI, magnetic resonance spectroscopic imaging; NAA, N‐acetyl aspartate; NMR, nuclear magnetic resonance; PC, pyruvate carboxylase; PDH, pyruvate dehydrogenase; PMG, primary microglia; PPP, pentose phosphate pathway; rCHI, repetitive closed head injury; TBI, traumatic brain injury; αKG, α‐ketoglutarate; β‐HB, β‐hydroxybutyrate.

**TABLE 3:** Summary of studies using ^13^C isotope tracing to assess metabolic response to TBI in human subjects.

Substrate	Tracer(s)	Study	*n*, TBI	*n*, ctrl	TBI severity	Tracer route	Sample analysis	Pathway(s) studied	Primary metabolite isotopomer(s) analyzed	Main conclusions
Glucose	1,2‐^13^C_2_‐glucose	Dusick et al. 2007^106^	6	6	Severe	IV	GC–MS	LDH & PPP	^13^C‐lactate	Flux of glucose into the PPP during the acute phase may indicate alternative intracellular energy allocation is necessary for preventing secondary injury
		Jalloh et al. 2015^129^	15	6	Severe	CMD	^13^C NMR & ^1^H NMR	Glycolysis, LDH, & PPP	^13^C‐lactate	While cerebral anaerobic metabolism is the major source of glucose‐derived lactate in the brain following TBI, glucose metabolism shifts to PPP as oxygen levels decrease
Lactate	3‐^13^C‐lactate	Glenn et al. 2014^143^	12	6	Moderate to severe	IV	GC–MS	GNG & TCA	^13^C‐glucose	GNG from peripheral glycogenolysis and lactate shuttling underlies post‐traumatic hyperglycemia to provide energy for vital organs including the brain
		Glenn et al. 2015^142^	12	6	Moderate to severe	IV	GC–MS	GNG & TCA	^13^CO_2_ & ^13^C‐glucose	Peripheral lactate is directly or indirectly the main source of carbohydrate substrate consumed by the brain following trauma
		Jalloh et al. 2018^141^	9	5	Severe	CMD	^13^C NMR & ^1^H NMR	TCA	^13^C‐glutamine	Cerebral oxidative metabolism of lactate is occasionally increased following TBI compared to non‐TBI controls, which may implicate its use as an emergency fuel source
	3‐^13^C‐lactate (w/2‐^13^C‐acetate)	Gallagher et al. 2009^62^	14	–	Severe	CMD	^13^C NMR	TCA	^13^C‐glutamine	First example of cerebral oxidative metabolism of lactate in human subjects
Pyruvate	1‐^13^C‐pyruvate	Hackett et al. 2020^55^	2	–	Mild	IV	MRSI	TCA & LDH	^13^C‐lactate & ^13^C‐bicarbonate	Established the use of ^13^C‐pyruvate metabolism using MRSI as a non‐invasive method for detecting altered cerebral metabolism in radiographically normal TBI subjects
Succinate	2,3‐^13^C_2_‐succinate	Jalloh et al. 2017^145^	9	–	Severe	CMD	^13^C NMR & ^1^H NMR	TCA & LDH	^13^C‐malate, ^13^C‐lactate, & ^13^C‐glutamine	Succinate administered via CMD supports ETC functioning by bypassing complex I, as indicated by reduced LPR and increased glucose utilization in TBI patients
		Khellaf et al. 2021^144^	36^a^	–	Mild to severe	CMD	^13^C NMR & ^1^H NMR	TCA & LDH	^13^C‐lactate & ^13^C‐pyruvate	Validated that clinical multimodal neurointensive monitoring can identify patients with mitochondrial dysfunction who may benefit from CMD succinate administration

Abbreviations: CMD, cerebral microdialysis; ctrl, healthy controls; ETC, electron transport chain; GC–MS, gas chromatography with mass spectrometry; GNG, gluconeogenesis; IV, intravenous; LDH, lactate dehydrogenase; LPR, lactate/pyruvate ratio; MD, microdialysis; MRSI, magnetic resonance spectroscopic imaging; NMR, nuclear magnetic resonance; PPP, pentose phosphate pathway; TBI, traumatic brain injury; TCA, tricarboxylic acid cycle.

^a^ Not all patients in cohort met clinical monitoring criteria to receive stable isotope administration.

**FIGURE 2:**
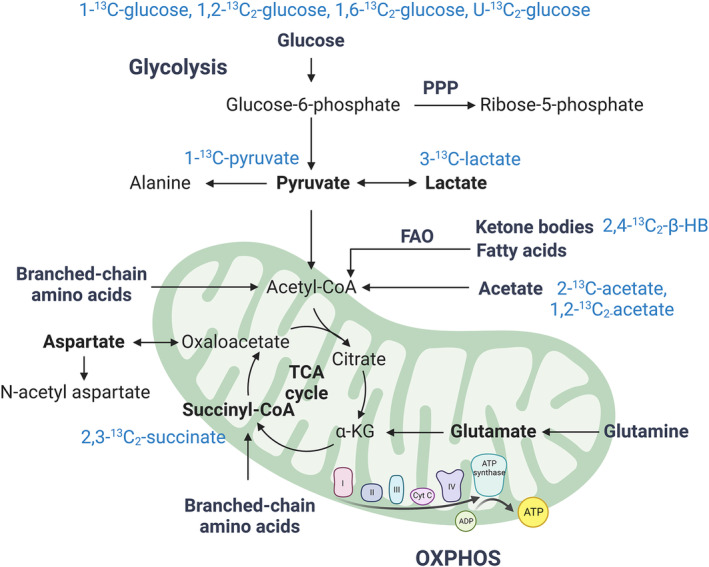
Schematic of fuel utilization and ^13^C‐isotope tracing in TBI studies. The metabolic flux of glucose, ketone bodies, fatty acids, acetate, glutamine, and branched‐chain amino acids into the TCA cycle is intricately linked with mitochondrial OXPHOS and ATP production. The reviewed ^13^C isotope tracers that have previously been employed to investigate metabolic flux in TBI are indicated in blue font. FAO, fatty acid oxidation; OXPHOS, oxidative phosphorylation; PPP, pentose phosphate pathway; TCA, tricarboxylic acid; αKG, α‐ketoglutarate; β‐HB, β‐hydroxybutyrate.

Because flux itself isn't measurable, isotope tracing takes advantage of exogenously introduced fuel sources (Figure 2) that are labeled with naturally occurring stable isotopes of key organic atoms which don't radioactively decay (e.g. ^1^H, ^13^C, ^15^N, or ^31^P). As the body consumes these substrates through various metabolic pathways, the movement of the isotopic atom is trackable because it is incorporated into metabolites at specific locations in a pathway‐dependent manner, each with a unique chemical signature that can be detected and quantified. Then, changes in the relative abundance of these metabolites over time can be used to infer overall flux through the said pathway. Strategic coupling of multiple tracers can infer complex metabolic activities, including glycolysis, TCA flux reversibility, gluconeogenesis, and other biosynthetic pathways.^11^

Signal detection and quantification in isotope tracing relies on various techniques, each with distinct advantages and limitations. NMR and LC/GC–MS are extensively used analytic techniques in isotope tracing studies in the current literature.^11^, ^12^, ^13^, ^33^, ^117^, ^118^, ^119^, ^120^ These established techniques provide detailed data on numerous substrates and metabolites in a sample. Recent computing advancements facilitate cross‐referencing metabolite readouts to libraries, aiding in identifying unexpected pathway alterations.^121^ In clinical settings, sample procurement for these methods is often via CMD catheters or central venous lines, which carry notable risks.^56^ However, these access routes are commonly used in intensive care for routine monitoring, potentially imposing minimal additional risk. In cases where NMR or LC/GC–MS sample collection is restricted, recent studies highlight MRSI as a non‐invasive and appealing alternative.^54^, ^122^ MRSI additionally provides spatial context to the quantitative data that may be useful for better understanding the global metabolic changes that occur in the brain following trauma, which the former detection methods typically lack. Therefore, the detection method should be carefully selected considering safety concerns, logistical constraints, and the overall objectives of the individual study employing isotope tracing.

### 4.1 ^13^C‐glucose tracing

**FIGURE 3:**
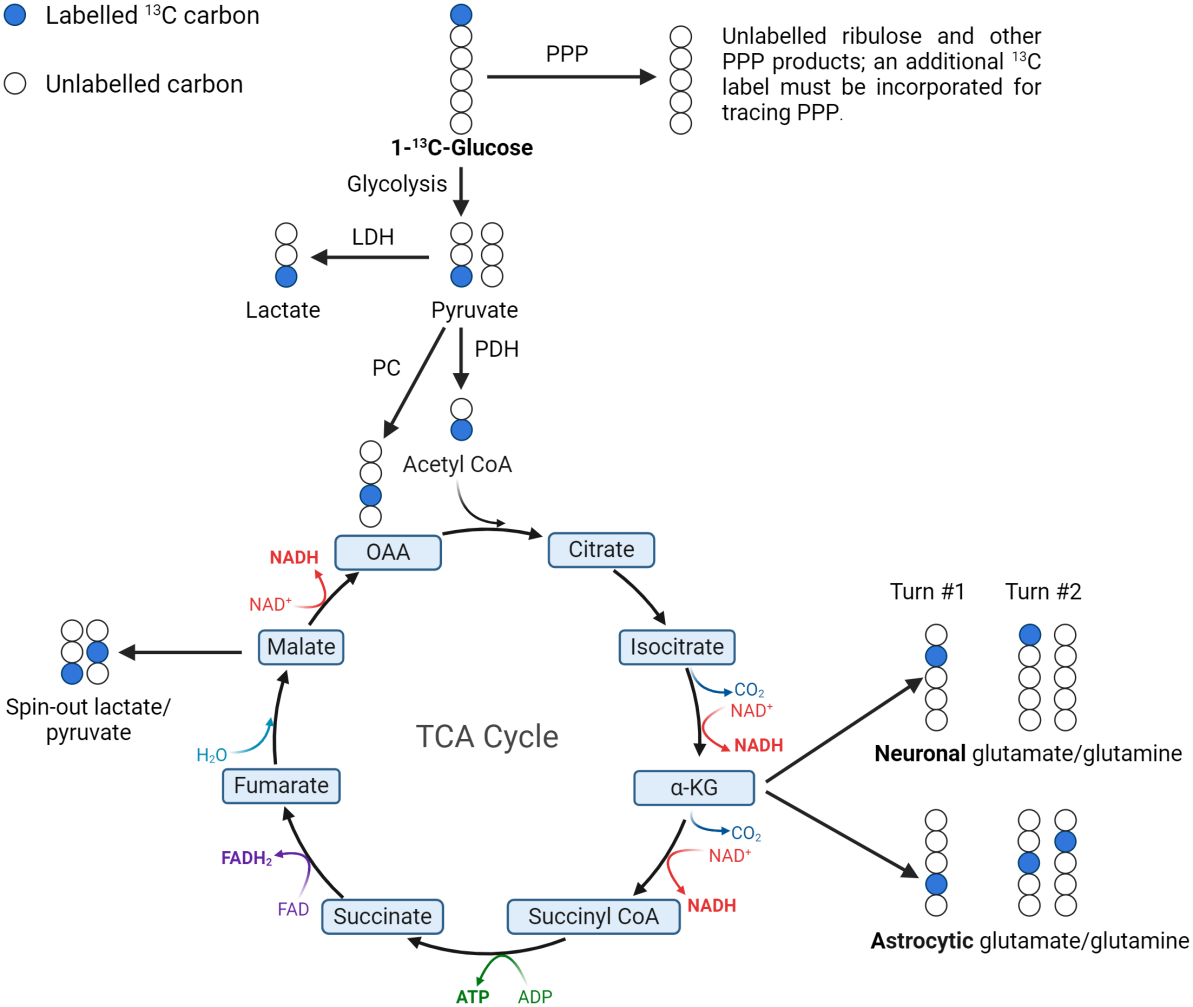
Labeling patterns for energetic metabolism of the tracer 1‐^13^C‐glucose. Labeled glucose may either be converted to pyruvate via glycolysis or shunted into the PPP. The produced labeled pyruvate may either be metabolized anaerobically via LDH or enter the TCA cycle via PDH (neuronal compartment) or PC (astrocytic compartment). OAA, oxaloacetate; PC, pyruvate carboxylase; PDH, pyruvate dehydrogenase; PPP, pentose phosphate pathway; TCA, tricarboxylic acid; αKG, α‐ketoglutarate.

^13^C‐labeled glucose tracers are widely employed in models^33^, ^41^, ^86^, ^123^, ^124^, ^125^, ^126^, ^127^, ^128^ and human studies^106^, ^129^ of metabolic dysfunction in TBI (Figure 2). One notable example is 1‐^13^C‐glucose, which allows accurate tracing of its single labeled carbon molecule through glycolysis and the TCA cycle (Figure 3). Due to innate differences in metabolic biochemistry in neurons versus astrocytes at several steps, this tracer is additionally able to partially discern contributions to overall cerebral glucose metabolism made by the astrocytic compartment. As such, it has proven useful for evaluating the interplay between anaerobic respiration and oxidative metabolism in the brain following trauma.^47^, ^86^, ^123^ However, because the C1 carbon of the glucose backbone is lost as CO_2_ in the hexose to pentose conversion step of the pentose phosphate pathway (PPP), which serves as a vital source of antioxidants for combating the increased ROS production in brain injury,^130^ this tracer is poorly suited to track PPP metabolic flux, a major limitation of its utility in TBI.

To circumvent this, glucose tracers with an additional labeled carbon, such as 1,2‐ and 1,6‐^13^C_2_‐glucose, may be employed. These tracers maintain the tracing capabilities of 1‐^13^C‐glucose through glycolysis and the TCA, while also enabling the investigation of PPP activity.^106^, ^124^, ^125^, ^126^, ^127^, ^128^, ^129^, ^131^ The addition of a labeled carbon in the C2 or C6 position allows for the detection of 1‐ or 5‐^13^C‐ribulose‐5‐phosphate signals when glucose enters the oxidative PPP, respectively. The movement of these metabolites can then be followed back into glycolysis via the production of monolabeled fructose‐6‐phosphate (e.g. 1‐^13^C‐fructose‐6‐phosphate) and subsequent downstream metabolites which aren't produced by glycolytic metabolism of dilabeled glucose.^11^, ^132^ Alternatively, the absence of this signature concurrent with reduced monolabeled ribulose‐5‐phosphate levels indicates the pentose products of the PPP are instead being used for nucleotide synthesis.^11^

Studies employing glucose tracers in TBI consistently validate established hypotheses regarding cerebral metabolomics after injury, confirming the decrease in oxidative glucose metabolism and subsequent acute hyperglycolytic phase during early secondary injury.^33^, ^41^, ^86^, ^123^, ^124^, ^125^, ^126^, ^127^, ^128^, ^129^ This technique also robustly supports the acute diversion of glucose into the PPP, supporting the theory inflammatory cascades induce early hyperglycolysis, causing significant ROS‐mediated damage and necessitating increased NADPH production for protection.^106^, ^125^, ^127^, ^129^, ^131^ Additionally, despite the initial hyperenergetic phase, a significant reduction in overall anaplerotic flux at 24 h, coupled with an increased pyruvate carboxylase/PDH ratio, implies a shift in glucose usage from energy production to regenerative processes later in secondary injury.^124^ Hence, glucose tracers play a crucial role in confirming and extending existing knowledge while identifying new directions for glucose‐mediated cerebral metabolomics research in TBI.

### 4.2 ^13^C‐pyruvate tracing

Labeled pyruvate enables the differentiation between oxidative and anaerobic consumption of glycolytic metabolites. It directly assesses pivotal enzyme activity, such as PDH, linking glycolysis to mitochondrial OXPHOS, and lactate dehydrogenase (LDH), a source of ATP production independent of mitochondrial respiration.^133^ Importantly, the cerebral extracellular LPR, a well‐studied measure in neurocritical care, consistently correlates with increased mortality and morbidity in TBI patients.^134^, ^135^ However, while valuable for prognosis, LPR alone doesn't capture the molecular conditions underlying altered lactate production or consumption, as discussed later.

The utilization of 1‐^13^C‐pyruvate has shed light on post‐TBI mechanisms^54^, ^55^, ^122^, ^136^, ^137^ (Figure 2), revealing how ^13^C‐bicarbonate production, an OXPHOS activity marker,^136^ aligns with ^13^C‐pyruvate mitochondrial consumption. Impaired mitochondrial function is marked by reduced bicarbonate and increased ^13^C‐lactate levels due to a shift toward anaerobic respiration via LDH. Studies in both animals and humans post‐TBI have consistently found significant mitochondrial dysfunction from the acute phase to at least a week post‐injury, necessitating a switch from oxidative to anaerobic respiration to meet cerebral energy needs.^54^, ^55^, ^122^, ^136^, ^137^ One study using hyperpolarized 1‐^13^C‐pyruvate demonstrated these changes are detectable in repetitive TBI brains extending beyond 3 months post‐injury.^137^ Others have utilized it to explore aspects of pyruvate metabolism in the injured brain in closer detail. For example, depleting microglia before TBI induction lessens pyruvate metabolism dysregulation, highlighting the significant role of microglial activation in these bioenergetic changes.^122^ Furthermore, dichloroacetate coadministration, a PDH kinase inhibitor, showed no difference in PDH activity, indicating aerobic metabolism reduction is not due to PDH kinase inhibition.^53^ Interestingly, all studies using 1‐^13^C‐pyruvate to date have employed MRSI as the detection method, highlighting its potential as an early, noninvasive imaging modality for identifying cerebral metabolic changes in otherwise radiographically normal TBI patients.^55^ Further, the use of such technology in tandem with machine learning workflows was recently shown to be capable of predicting long‐term behavior changes after TBI in animals that otherwise lack detectable changes in conventional imaging at the same timepoint,^137^ which suggests isotope tracing may be a powerful approach to better study the link between TBI and the elevated risk of developing long‐term neurobehavioral sequelae including mental health disorders^138^ and dementia,^139^ among others. However, it is critical to note that the application of hyperpolarized 1‐^13^C‐pyruvate in TBI models has shown a rapid lactate increase at the injury site,^136^ potentially worsening the focal injury and posing limitations for its use in TBI patient studies, and this must be carefully considered when designing experiments or clinical procedures.

### 4.3 ^13^C‐lactate tracing

Lactate serves as a viable neuron fuel and participates in intricate homeostatic interactions between excitatory neurons and astrocytes.^140^ Recently, clinical studies using labeled lactate have sought to better understand neurons and astrocytes activity following trauma and its relation to patient prognosis.^62^, ^141^, ^142^ A study using 3‐^13^C‐lactate tracing was the first to demonstrate the brain's capability of using lactate as a fuel source following head trauma^62^ and it was later shown that lactate is utilized differently in the post‐traumatic brain compared to controls in humans^141^ (Figure 2). This indicates situational lactate metabolism can compensate for the reduced glucose processing capability of the brain in the depressed metabolic phase of secondary injury. Further, additional studies have demonstrated peripheral lactate mobilization is at least partly responsible for the systemic hyperglycemia that is known to occur following trauma^143^ and that lactate‐derived carbohydrate substrates either directly, via oxidative metabolism of lactate, or indirectly, via peripheral gluconeogenesis, provide the majority of metabolic energy to the brain after TBI.^142^ Nevertheless, severe TBI can render neurons incapable of using lactate as an alternate fuel source to meet metabolic needs, which can focally elevate lactate concentrations to a degree that may damage or even kill local tissues.^41^ Hence, lactate's role in the brain in health and injury is complex, and future lactate tracing studies can directly probe its function as both a waste product and fuel source.

### 4.4 ^13^C‐β‐hydroxybutyrate and succinate tracing

Several additional tracers have been used in the setting of TBI.^84^, ^144^, ^145^ As PDH may be directly inhibited by ROS, PDH dysfunction is one of the commonest causes of mitochondrial dysfunction and subsequent OXPHOS impairment in trauma.^146^ The ketone body β‐hydroxybutyrate (β‐HB) can be directly converted to acetyl‐CoA, independent of PDH activity, making labeled β‐HB suitable for assessing mitochondrial enzyme activities within the TCA cycle.^84^ In a TBI model, 2,4‐^13^C_2_‐β‐HB tracer revealed increased ketone metabolism enhancing TCA and subsequent OXPHOS activity in both neurons and astrocytes despite PDH dysfunction^84^ (Figure 2). Succinate metabolism via succinate dehydrogenase provides electrons to the electron transport chain (ETC) independently of NADH reduction via mitochondrial complex I.^147^ Focal 2,3‐^13^C_2_‐succinate administration improved cerebral TCA functioning by bypassing complex I, supporting ETC activity.^144^, ^145^ While the impact on patient outcomes remains uncertain, these studies offer unique insights into oxidative glucose metabolism dysfunction post‐injury and warrants additional studies.

### 4.5 Combination tracing

Combination of multiple labeled tracers can enhance understanding of cerebral bioenergetic dysregulation in TBI, elucidating contributions from distinct cell populations and specific metabolic aspects. Co‐administering 1‐^13^C‐glucose and 1,2‐^13^C_2_‐acetate in experimental TBI revealed greater reductions in oxidative glucose metabolism in neurons than astrocytes (Figure 2). Astrocytes, in response to TBI, utilize acetate to elevate glutamate concentrations through the glutamate‐glutamine cycle, attempting to restore metabolic function post‐injury.^86^ By comparing TCA metabolite labeling patterns from animals given either 1‐^13^C‐glucose, 3‐^13^C‐lactate, or 2‐^13^C‐acetate, it was further shown neuronal metabolic dysfunction becomes entirely uncoupled from the metabolic activity of the astrocytic compartment within minutes of severe TBI, demonstrating that astrocytic lactate production continues despite impaired neuronal consumption of the substrate.^41^ These examples highlight only a few examples of how tracer combinations can unravel complex metabolic changes and compartmental contributions in secondary injury. Selecting novel tracer combinations for future investigations holds promise in advancing our understanding of TBI.

## 5 CONCLUSIONS AND PERSPECTIVE

^13^C isotope tracing emerges as a powerful technique for analyzing altered metabolic flux and unraveling the complex molecular underpinnings of secondary brain injury. Various ^13^C‐labeled tracers have successfully illuminated diverse facets of TBI metabolism in both animal models and human subjects. Particularly in animal studies, ^13^C isotope tracing has enabled more invasive and detailed metabolic analyses, such as evaluating metabolism and enzyme activity within specific brain regions, enabled by post‐mortem analyses. These insights are foundational for generating hypotheses and decoding fundamental metabolic mechanisms. However, applying these insights to human contexts necessitates careful navigation of the differences in metabolism and physiology between species.

The link between early mitochondrial dysfunction and bioenergetic metabolism dysregulation with TBI severity underscores the importance of recognizing that metabolic profiles can significantly vary among mild, moderate, and severe TBI cases, evolving across different stages post‐injury. Investigating these connections is essential, as it may lead to groundbreaking diagnostic and therapeutic strategies for TBI. For instance, mild TBI might exhibit only transient mitochondrial disturbances and metabolic shifts, which can often be overcome with minimal intervention. However, moderate to severe TBI are likely to experience more prolonged and pronounced mitochondrial dysfunction and metabolic dysregulation, potentially resulting in an extended energy crisis that contributes to continued cellular damage and a prolonged recovery process. Although long‐term mental health problems post‐TBI are well‐documented,^148^ predicting these outcomes with conventional techniques like CT or MRI remains challenging. Utilizing ^13^C tracer in conjunction with advanced imaging methods such as PET or MRS to monitor specific metabolic derangements and their thresholds could offer valuable prognostic information, enabling clinicians to assess the risk of poor outcomes, including long‐term cognitive impairment, especially in patients with mTBI. For example, a recent study has employed hyperpolarized 1‐^13^C pyruvate with MRSI to visualize acute metabolic changes in mild TBI patients.^55^

Tracing technologies have proven indispensable in dissecting the complex pathophysiology of TBI. Unlike conventional metabolic assessment techniques that rely on standard laboratory equipment, methods like NMR and LC/GC–MS for quantifying ^13^C isotopic metabolite signals are not universally accessible and demand intricate computational analysis post‐data collection. Moreover, tracer technologies, dependent on metabolic pathway differences specific to cell types, struggle to differentiate activities across various cell populations in vivo, a limitation also found in traditional metabolic techniques. Overcoming this challenge may be possible through complementary in vitro studies^33^ or by isolating brain cells post‐TBI following ^13^C isotope infusion. Yet, relying solely on ^13^C isotopes may not completely reveal the metabolic processes behind the secondary injuries of TBI. Other tracers, each with unique benefits for dissecting TBI pathology, complement the capabilities of ^13^C isotope tracer. For instance, ^18^F‐fluorodeoxyglucose offers insights into glucose metabolism,^149^ while ^11^C‐Pittsburgh compound B targets amyloid deposits, illuminating post‐TBI neurodegenerative processes.^150^ Integrating ^13^C with other isotope tracers like ^1^H, ^15^N, or ^31^P, commonly utilized in TBI studies across humans and animals, could enable a more thorough investigation of cerebral metabolic disturbances.^60^ Furthermore, the development and application of novel ^13^C tracers remain crucial for advancing our understanding of post‐trauma cerebral metabolic dysregulation. For instance, using ^13^C octanoate, a BBB‐permeable medium‐chain FA constituting a significant portion of the normal free FA pool, could offer insights into brain FA metabolism.^151^ Employing ^13^C isotope tracing for amino acids such as glutamine, aspartate, and branched‐chain amino acids will deepen our comprehension of amino acid metabolism in TBI.^12^

Exploring new therapeutic avenues for TBI through ^13^C isotope tracing, by elucidating specific metabolic pathways and dysfunctions, could identify novel therapeutic targets. Pinpointing areas of mitochondrial dysfunction or abnormal glucose, amino acids, and fatty acids metabolism may lead to interventions designed to restore metabolic balance and mitigate secondary injury.^152^ Additionally, ^13^C tracing technologies may facilitate personalized treatment strategies, enabling clinicians to customize interventions based on individual metabolic profiles, potentially enhancing outcomes. The application of isotope tracing may also provide a method to evaluate the efficacy of therapeutic interventions in real time, with changes in tracer uptake or metabolite levels signaling whether a treatment is effectively addressing metabolic dysfunction or improving cerebral blood flow post‐TBI.^153^, ^154^

In summary, the application of ^13^C isotope tracing is poised to significantly enhance our understanding of the metabolic mechanisms underpinning secondary injuries in TBI, contributing to the development of novel diagnostic tools, techniques, and therapeutic targets. Moreover, isotope tracing paired with MRSI or other analytic in vivo imaging techniques has the potential to more accurately identify and characterize patients with TBI in the clinical setting, ultimately improving their quality of care and outcomes.

## AUTHOR CONTRIBUTIONS

CJP, MDK, and NL created the original draft of the manuscript. CJP, MDK, and NL generated tables and figures. CJP, MDK, YJ, YX, WX, YW, MS, DZ, ASD, XW, and NL critically revised the manuscript. ASD, XW, and NL supervised the project. All authors have read and approved the final version of the manuscript prior to submission.

## FUNDING INFORMATION

National Institutes of Health (NIH) grants (1RO1 NS126503‐01A1 to XW), American Heart Association Career Development Award (23CDA1055341 to NL), A pilot funding (to NL) from the Tulane COBRE for Clinical and Translational Research in Cardiometabolic Diseases, National Institutes of Health (P20GM109036), and Tulane School of Medicine Pilot Fund Award.

## CONFLICT OF INTEREST STATEMENT

Dr. Xiaoying Wang is an Editorial Board member of *CNS Neuroscience and Therapeutics* and a co‐author of this article. To minimize bias, he was excluded from all editorial decision‐making related to the acceptance of this article for publication. The authors declared no potential conflicts of interest with respect to the authorship of this article.

## Data Availability

Data sharing is not applicable to this article as no new data were created or analyzed in this study.
